# The correlation between the margin of resection and prognosis in esophagogastric junction adenocarcinoma

**DOI:** 10.1186/s12957-023-03202-7

**Published:** 2023-10-09

**Authors:** Tao Pang, Mingming Nie, Kai Yin

**Affiliations:** Department of Gastrointestinal Tract Surgery, First Affiliated Hospital of Naval Military Medical University, Shanghai, China

**Keywords:** Esophagogastric junction adenocarcinoma, Proximity margin, Surgical approaches, Neoadjuvant therapy, Signet ring cells

## Abstract

Adenocarcinoma of the gastroesophageal junction (AEG) has become increasingly common in Western and Asian populations. Surgical resection is the mainstay of treatment for AEG; however, determining the distance from the upper edge of the tumor to the esophageal margin (PM) is essential for accurate prognosis. Despite the relevance of these studies, most have been retrospective and vary widely in their conclusions. The PM is now widely accepted to have an impact on patient outcomes but can be masked by TNM at later stages. Extended PM is associated with improved outcomes, but the optimal PM is uncertain. Academics continue to debate the surgical route, extent of lymphadenectomy, preoperative tumor size assessment, intraoperative cryosection, neoadjuvant therapy, and other aspects to further ensure a negative margin in patients with gastroesophageal adenocarcinoma. This review summarizes and evaluates the findings from these studies and suggests that the choice of approach for patients with adenocarcinoma of the esophagogastric junction should take into account the extent of esophagectomy and lymphadenectomy. Although several guidelines and reviews recommend the routine use of intraoperative cryosections to evaluate surgical margins, its generalizability is limited. Furthermore, neoadjuvant chemotherapy and radiotherapy are more likely to increase the R0 resection rate. In particular, intraoperative cryosections and neoadjuvant chemoradiotherapy were found to be more effective for achieving negative resection margins in signet ring cell carcinoma.

Adenocarcinoma of the gastroesophageal junction (AEG) has become more prevalent in Western and Asian populations, and surgical resection is currently the primary treatment. However, there is still no consensus on its origin and distinctive biological features [1], making it difficult to establish standardized therapeutic strategies, including perioperative treatment, surgical approach, lymph node dissection, and the extent of esophageal resection. An essential factor that affects treatment strategy is the length of the proximal margin (PM), which is the distance from the upper edge of the tumor to the esophageal margin. The debate over the impact of the length of the PM on prognosis is ongoing in the field of gastrointestinal surgery, and there is currently no consensus. As AEG affects both the esophagus and stomach, it poses unique challenges in surgical management. The extent of the esophageal resection and lymph node dissection should be carefully considered. Additionally, the use of neoadjuvant therapy, intraoperative cryosection, and other techniques may play a role in achieving negative resection margins. In conclusion, the length of the PM is a crucial factor in determining prognosis and treatment strategy [2].

This article aims investigate the impact of PM on the prognosis of patients diagnosed with gastroesophageal adenocarcinoma from multiple perspectives. Specifically, it will discuss the optimal length around the proximal resection margin and its impact on patient outcomes, as well as how to ensure optimal PM from two different perspectives.

## The impact of esophageal margin on the prognosis of AEG patients

The postoperative surgical esophageal margin status is an important prognostic factor in AEG. R0 margin, free of visible tumor cells under the microscope, is associated with a better prognosis, while the presence of tumor cells at or near the margin (R1/R2), is associated with a higher risk of recurrence and poorer outcomes. However, the relationship between the margin status and prognosis is complex and can depend on several factors, such as tumor size, location, stage, and patient characteristics, as well as the specific surgical technique used. In addition, other factors, such as the presence of lymph node metastases and the completeness of the resection, also play an important role in determining the overall prognosis. In the case of AEG, the relationship between the margin and prognosis primarily revolves around two key issues.

### Does R0 resection of the esophageal margin versus R1/R2 resection affect the prognosis of patients?

In most studies, positive margins (R1/R2) are associated with lower survival rates [3–6], regardless of the surgical approach or tumor location. Achieving an R0 resection has been shown to increase median overall survival by 8–25 months following surgery [4, 6]. However, some studies have suggested that positive margins may not be an independent prognostic factor for AEG survival [7, 8]. For instance, DiMusto et al. found that 80% of AEG patients with positive margins ultimately developed distant metastases and that more extensive esophageal resection or additional treatment for positive margins did not improve prognosis or reduce local recurrence [9]. These findings may be due, in part, to the fact that TNM staging can obscure the effect of positive margins, and advanced-stage tumors are less likely to be completely removed and achieve R0 resection [10]. Therefore, it remains a challenge to determine whether to pursue R0 margins and which stage of AEG should be achieving R0 resection.

The National Cancer Database (NCDB) in the USA [11] reports that out of 3125 AEG patients with pT1-3N0-1M0, 342 (10.9%) had positive margins, with 218 (7%) undergoing R1 resection and 124 (4%) undergoing R2 resection. The study revealed that increasing clinical T status was an independent predictor of positive margins. Interestingly, lymph node N status did not predict the occurrence of positive margins. Importantly, the study also found that patients with positive margins had significantly lower 5-year survival rates compared to those with negative margins.

Bickenbach's study yielded comparable findings, indicating that positive margins were linked to reduced survival rates among T1–T2 patients or those with less than three positive lymph nodes, but not among patients with more advanced stages [12]. During surgery, it is often difficult to determine the exact number of positive lymph nodes. Typically, we must rely on judgments made from frozen sections of the margin. Unfortunately, the impact of margins on prognosis can become obscured as the TNM stage advances [13, 14]. Patients with later-stage TNM are more likely to have positive margins, further complicating the situation. These AEGs exhibit greater invasiveness, which means that even if the surgical margins are negative, large and poorly differentiated tumors may still spread beyond the surgical range. As a result, surgery alone may not be sufficient to cure these patients. Additionally, this can lead to similar survival rates for patients regardless of whether they receive false-negative or true-positive margin results (Table 1) [15].

**Table 1:** Prognostic impact of esophageal-positive margins in different TNM stages

Study	*N*	*N*(positive)	T stage	N stage	Positive vs negative margins
DiMusto et al. [9]	1044	20			
Javidfar et al. [11]	3125	342	T1–3^a^	≤ 2^b^	*p* < 0.01
Bickenbach et al. [12]	2384	108	T1–2^a^	< 3^b^	*p* < 0.01
Zhe Sun et al. [13]	2159	110	T1–2^a^	≤ 2^b^	*p* < 0.01
Cho BC et al. [14]	2740	49	T2^a^	0^b^	*p* < 0.01
Nagata T et al. [16]	1205	23	/	/	*p* < 0.01
Wang et al. [17]	1565	129	T1–2^a^	≤ 2^b^	*p* < 0.01

^a^Statistical differences in positive margins between different T stages

^b^Statistical differences in positive margins between different N stages

Based on the available studies, it appears that patients who undergo R0 resection have a 5-year survival rate ranging from 53 to 60%, whereas those who undergo R1 resection have a much lower survival rate of 13% to 26% [13, 16–18]. Also, we can temporarily think that the incidence of a positive margin is lower in T1-2N0-2 AEG, but a positive margin has a significant impact on prognosis. With the continuous progress of staging, the impact of a positive margin on prognosis after T3-4N3 will gradually weaken, but a negative margin should be ensured at this time.

Research has demonstrated that prolonged esophagectomy leads to higher survival rates, as patients have been found to survive for an average of 37 months. This is in contrast to the average survival time of 22 months for patients who undergo standard gastrectomy [19]. Although postoperative chemotherapy or chemoradiotherapy (D2 gastrectomy) may extend disease-free survival (DFS), the primary objective of surgery is to attain R0 resection, regardless of other factors and the type of surgery performed [20, 21]. This raises the question of whether a longer PM duration for patients with locally advanced AEG could enhance their prognosis.

### Is a longer distance from the PM associated with a better prognosis? And how can the optimal distance for a PM be determined?

In this subject, we aim to explore whether increasing the length of the PM can improve the prognosis of AEG patients who undergo surgery with the goal of achieving R0 resection. If this is the case, we also seek to determine the minimum length of the PM required to achieve this outcome. However, we note that there is currently no universally accepted standard for evaluating the distance from the surgical margin, nor is there a consensus on when to evaluate it. As a result, the selection of PM length is often based on the tradition of the medical institution or the preference of the surgeon, rather than on evidence-based data [22]. During surgery, surgeons typically assess the surgical margin by visually examining and feeling it to determine if the resection is sufficient. However, of the 13 studies conducted on PM issues, only five have established a minimum length for the PM. Based on current research, a gross PM length of 5–12 cm is necessary to ensure that no tumor residue [4, 19, 23–25].

According to the report by Papachristou et al. [23], it was observed that only esophagi that were macroscopically tumor-free and had undergone a resection of 12 cm or more were able to avoid positive proximal margins. Additionally, the study found that once the tumor had invaded the esophagus by more than 4 cm, the incidence of intramural skip metastasis significantly increased. The study also found that patients with a longer PM had a higher survival rate after surgery, indicating that the length of the PM should be taken into consideration when determining the best treatment approach for AEG patients. However, an increasing length of the esophagus would require thoracotomy, and a high risk of complications would arise from this procedure. Therefore, clinical guidance for AEG patients remains limited.

The study from Memorial Sloan-Kettering Cancer Center revealed that the extended esophagectomy group (proximal stomach and esophagogastric anastomosis in the chest (Ivor Lewis operation) or neck (3-phase operation); or via transhiatal approach with anastomosis in the neck) had a higher 5-year survival rate of 37%, compared to the restrictive esophagectomy group's (including intra-abdominal esophagogastric anastomosis, total gastrectomy and thoracoabdominal procedures) 27%, indicating a statistical significance difference [19]. Further analysis found that there was a statistically significant difference between patients with a PM length of 3.8 cm and better prognosis with stage T2-4N0-2 postoperative pathological specimens (HR 0.45, *p* < 0.01). However, the survival rate of T2-4 AEG patients with stage N3 was not significantly affected by PM length. The margin length used in this study was measured on specimens stretched after formalin fixation and fixed onto cardboard, and fresh in situ esophagus contracted to 27% after fixation [26]. Hence, it has been determined that a minimum in situ margin length of 5 cm is necessary for improved survival in patients who undergo surgery alone, as opposed to the previously believed 3.8 cm. It is worth noting that the N staging utilized in this study was derived from the 6th edition of the AJCC staging system, which underwent revisions in the 7th edition, specifically regarding the definition of N2 and N3. As a result, patients who exhibit positive lymph nodes up to 6 (N0-2) should have their esophageal resection range extended as much as possible. There is a further issue arises, as it is challenging to ascertain the number of positive lymph nodes either before or during the operation. This makes it difficult to determine whether to extend the length of the esophageal resection. Consequently, it is necessary to explore more convenient and appropriate preoperative assessment methods. Based on this information, the surgical objective for AEG patients should be an R0 resection with margins greater than 5 cm above the tumor. Despite this, the 5cm distance may still pose a challenge for the transabdominal/transdiaphragmatic approach and could potentially be the maximum length of the esophagus that can be resected using this method.

In 2013, Mine conducted a study on 100 patients with AEG who had undergone intra-abdominal surgery and had a staging of pT2-4N0-3M0. The study found that 14% of the samples showed positive proximal margins, and PM > 2 cm after intra-abdominal surgery showed a more significant difference in survival compared to tumors with a proximal margin ≤ 2 cm (*P* = 0.027) [27]. The study revealed a remarkably low incidence of tumor recurrence in positive proximal margin, with only 1.4% of patients encountering anastomotic recurrence, which was no statistically significant difference when compared with patients with negative margins. It is worth noting that the 2 cm distance in the pathological specimen equates to 2.8 cm in the body. This distance is more acceptable to gastrointestinal surgeons when compared to the 5 cm distance in the Barbour study. In fact, it is currently the standard for ensuring the length of the esophageal margin in clinical practice. This conclusion is similar to that of Tsuruga et al. [24] who found that R0 resection was effective in patients with an infiltrating AEG with a PM greater than 2 cm. PM greater than 4 cm is more appropriate for AEG cases in which esophageal involvement is poor. The study employed the seventh edition of the AJCC staging system. The conclusion to extend the PM length based on the N stage aligns with the findings of the Barbour study.

In 2016, Bissolati et al. [28] conducted additional research on the correlation between PM and positive margins, concluding that the minimum PM should be 2 cm. Further stratified analysis revealed that in T1 patients, the sole determining risk factor for margin involvement was a PM < 2 cm. However, in T2-4 Lauren intestinal type AEG patients, a PM < 3 cm became an independent risk factor for margin involvement. In T2-4 Lauren diffuse/mixed type AEG patients, the average distance of PM was only 3.3 cm. The actual tumor infiltration length could reach up to 5.1 cm or even 12 cm, which aligns with the findings of Papachristou’s study. Determining a reliable and safe PM in this case appears to be a challenging task. The incidence of positive margins is higher in Lauren diffuse/mixed type AEG, leading the authors to suggest that intraoperative frozen section (IFS) should be widely utilized in T2-4 diffuse cancer. This recommendation is especially important when confirmed independent risk factors are present, including AEG tumor location (OR 2.8), serosal infiltration (OR 2.2), tumor size > 4 cm (OR 3.5), and lymphatic infiltration (OR 4.2).

In a different large-scale retrospective study [29], it was found that there was no significant correlation between the length of PM and survival in Siewert II/III AEG patients. This study had a sample size of 693 cases, which is notably larger than both Mine’s (100 cases) and Barbour’s (275 cases) studies. The study found a positive proximal margin incidence of approximately 2.0%, which is comparable to Barbour’s reported rate of 3.0% in 2007 and Mine’s reported rate of 1.4% in 2013. However, it is important to note that this study did not include tumors that invaded the lower esophagus by more than 3 cm. Additionally, all surgeries were performed via the diaphragmatic approach, with a limited range of lower esophageal resection and a proximal margin range of 0–5 cm. Out of the total cases, 207 (29.9%) were classified as N3 while 380 (54.8%) were categorized as stage III. Unfortunately, an early stratification analysis was not conducted, which could have impacted the accuracy of determining the correlation between PM length and survival.

According to the findings of this study, the 5th edition of the Japanese Gastric Cancer Association guidelines, which were published in 2018, suggested that advanced AEG patients should have a minimum PM of at least 3 cm for Borrmann types I/II and 5 cm for Borrmann types III/IV [30]. These values have not been altered in the 6th edition of the guidelines. The Dutch guidelines suggest a minimum PM of 6 cm, whereas the German guidelines propose 5 cm for intestinal-type AEG and 8 cm for diffuse gastric cancer [31]. It seems that the choice of PM in AEG is affected by several factors, including tumor staging and classification. Further clarification of these factors would be beneficial in determining the appropriate PM during surgery (Table 2).

**Table 2:** The minimum PM at different stages in each study and guidelines

Study	Intestinal type								Diffuse/mixed type							
	T1	T2	T3	T4	N0	N1	N2	N3	T1	T2	T3	T4	N0	N1	N2	N3
Papachristou et al. [23]																
Barbour et al. [19]		≥ 3.8 cm(5 cm in situ)						/		≥ 3.8 cm(5 cm in situ)						/
Mine et al. [27]		> 2 cm(2.8 cm in situ)								> 2 cm(2.8 cm in situ)						
Bissolati et al. [28]	≥ 2 cm	≥ 3 cm							≥ 2 cm	≥ 3.3 cm						
Tsujitani et al.’s tudy [24]	2 cm															
Feng F et al.’s study [29]	/	/			/				/	/			/			
Ito et al’s study [25]	≥ 4 cm	≥ 6 cm							≥ 4 cm	≥ 6 cm						
Japanese [32]	≥ 2 cm	≥ 3 cm (I/II)							≥ 2cm	≥ 5 cm (III/IV)						
Dutch [31]	≥ 6 cm								≥ 6 cm							
German [31]	≥ 5 cm								≥ 8 cm							

Based on current data and guidelines, it is recommended that the PM have a minimum margin of 2–6 cm and a total length of 4–12 cm. The total distance required for the PM varies depending on the stage of the tumor. These studies are still based mainly on retrospective studies with a low level of evidence. Therefore, it may be unsafe to use a 2-cm PM for AEG [33]. Currently, the definition of PM is not very accurate, and further prospective studies are needed to obtain reliable evidence for the optimal PM.

The current definition of oligometastasis in gastric cancer refers to a tumor that falls between being localized and metastatic, and has distinct biological features [34]. In cases where the length of esophageal invasion reaches a certain point, failure to achieve R0 resection or extensive lymph node metastasis can be considered oligometastasis of the gastroesophageal junction. In such cases, the recommended therapeutic strategy is localized treatment with systemic therapy. Of course, this length remains to be defined, and its biological characteristics will need to be further investigated (Table 3).

**Table 3:** Study design, CEBM level of evidence, and proximal margin measurement

Study	*N*	Study design	CEBM level of evidence	Proximal margin measurement
Papachristou et al. [23]	101	Retrospective	4	Macroscopic, before fixation
Barbour et al. [19]	505	Retrospective	4	Macroscopic, before fixation
Mine et al. [27]	140	Retrospective	4	Macroscopic, before fixation
Bissolati et al. [28]	191	Retrospective	4	Microscopic, after fixation
Tsujitani et al.’s study [24]	175	Retrospective	4	Macroscopic, before fixation
Feng F et al.’s study [29]	693	Retrospective	4	Macroscopic, before fixation
Mariette et al.’s study [35]	94	Retrospective	4	Macroscopic, before fixation
Ito et al.’s study [25]	82	Retrospective	4	Macroscopic, before fixation

## What approaches can we use to guarantee negative resection margins in the esophagus for patients with adenocarcinoma of the gastroesophageal junction?

To ensure a negative esophageal margin, various procedures were employed. These included thoracotomy for prolonged esophagectomy, intraoperative cryopreservation of the sections, and neoadjuvant therapy reducing stage to achieve R0 resection.

### Ensuring negative margins through different surgical approaches to improve patient prognosis

**Fig. 1:**
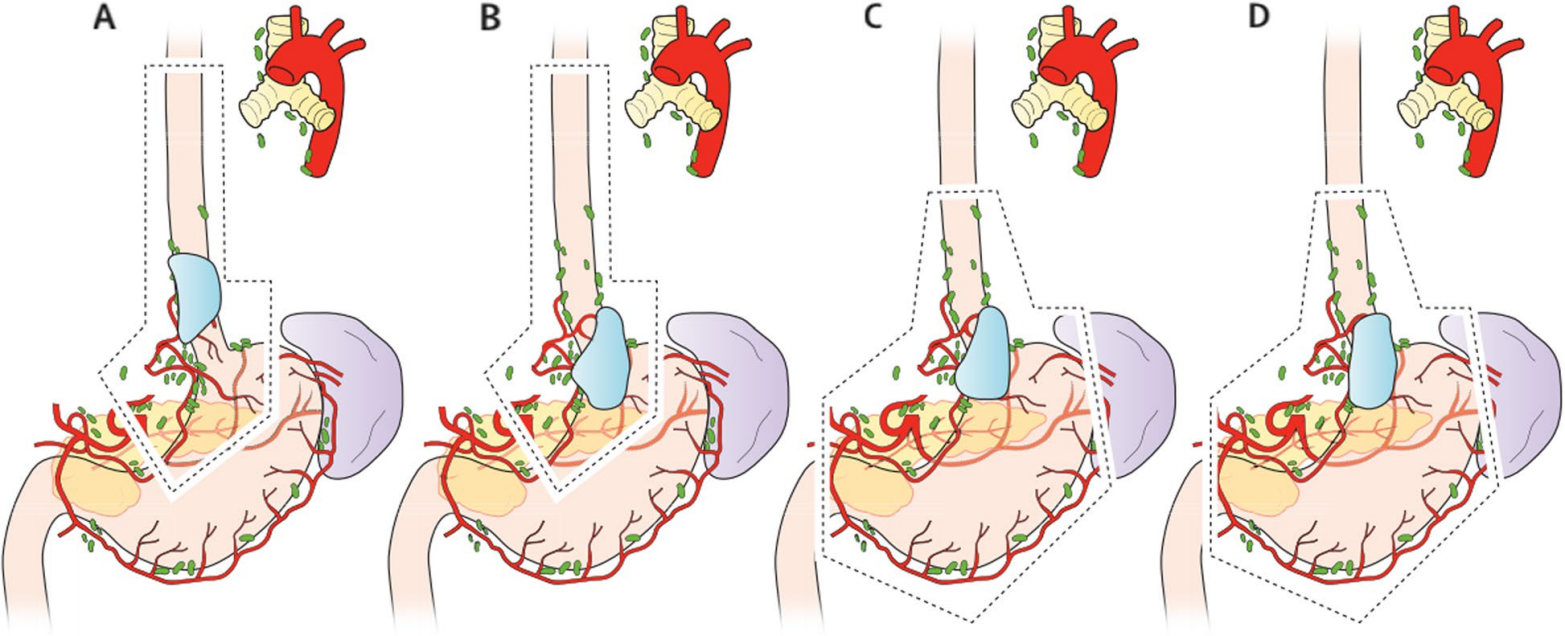
Schematic representation of the recommended extent of surgical resection for oesophagogastric junction adenocarcinomas [35]. Seiwert I type (**A** subtotal oesophagectomy with superior polar gastrectomy), Seiwert II type (subtotal oesophagectomy with superior polar gastrectomy (**B**) or total gastrectomy with inferior oesophagectomy (**C**)), and Seiwert III type (**D**; total gastrectomy). Blue region is the tumor site

Based on studies examining the impact of margin status on prognosis and recommended post-surgery treatment, it has been determined that laparotomy surgery solely on the abdominal segment of the esophagus is inadequate to meet the recommended treatment plan. This has led to ongoing debate among gastrointestinal and thoracic surgeons regarding the preferred approach, whether it be abdominal (diaphragmatic) or thoracic. In recent years, there have been comparative studies on thoracic and diaphragmatic approaches for Siewert type II adenocarcinoma of the esophagogastric junction (AEG) [35]. However, there is still no consensus on the optimal surgical approach for this condition. To reach an agreement, more precise preoperative assessments are needed, including determining the length of esophageal involvement, identifying the optimal perigastric lymph node management, and evaluating the value of lymph node dissection around the esophagus. The main goal is still to achieve complete (R0) tumor resection. Previous studies on the choice of surgical approach have focused on two aspects: (1) increasing the range of esophageal resection to obtain longer PM and improve patient survival; and (2) whether the different approaches result in differences in lymph node dissection and affect patient prognosis (Fig. 1).

In a study of Siewert I–III type AEG tumors, two surgical methods were compared: esophagectomy and extended gastrectomy. The study found that there were no significant differences in 30-day mortality or 5-year survival rates between the two methods [36]. Similarly, another retrospective study also failed to identify any statistically significant survival benefits associated with either surgical approach [37]. The reason for this is that esophagectomy is primarily utilized for type I AEG tumors, while extended gastrectomy is reserved for type II/III AEG tumors. Additionally, there is no comparison of PM extension following esophageal segment extended resection, which limits our ability to fully understand the impact of the surgical approach on prognosis.

A prospective randomized controlled trial was conducted by a Dutch research group to compare the effectiveness of right thoracotomy and diaphragmatic approaches in treating Siewert I/II tumors. The results showed that there was no significant difference in prognosis between the two approaches. Therefore, it is not recommended to use thoracotomy for Siewert II tumors [38]. Wei et al. [39]conducted a meta-analysis that revealed no significant difference in overall survival rates between thoracotomy and diaphragmatic approaches for Siewert II AEG. However, the diaphragmatic approach was found to reduce hospitalization time for pulmonary complications. In 2014, Haverkamp et al. conducted a systematic review of papers published between 1995 and 2013 on surgical strategies for AEG adenocarcinoma. Their findings showed no significant differences in benefits between esophageal expansion resection and extended gastric resection [40]. In Japan, a prospective randomized controlled trial was conducted to compare the left thoracoabdominal and diaphragmatic approaches for resection of Siewert II/III tumors with esophageal invasion of 3 cm or less. The study revealed that the left thoracoabdominal approach did not improve survival rates and would increase postoperative morbidity. However, this approach could completely remove the lymph nodes below the mediastinum [41]. According to previous research [29], lymph node metastasis is the only independent prognostic predictor for Siewert II/III AEG patients with esophageal invasion of 3 cm or less. The approach of lengthy PM does not seem to affect the prognosis of Siewert II/III AEG patients. This implies that for Siewert II/III AEG patients with esophageal invasion of 3 cm or less, abdominal (diaphragmatic) surgery alone can achieve adequate resection and improve prognosis. Therefore, it is recommended [42]. This also indicates that when the PM reaches a certain length, it will not affect the prognosis of patients, so the main reference basis for thoracotomy should be the optimal PM.

On the other side, the prognostic impact of peri-esophageal lymph node metastasis should be considered. In Siewert II tumors with an esophageal invasion greater than 3 cm, there is a higher likelihood of lymph node metastasis in the lower mediastinum [43]. In such instances, a thoracic approach with mediastinal lymph node dissection may offer therapeutic benefits [44]. The Siewert study found that only 15% of type II/III AEG patients had positive lymph nodes near the esophagus [36]. However, it is important to note that the study only included patients who underwent a diaphragmatic approach, which limited their lymph node dissection to D2 of the lower mediastinum and abdominal lymph nodes. The study did not include lymph node dissection or pathological evaluation of higher mediastinal lymph nodes. As a result, it is unclear whether type II patients may have lymph node metastasis in any direction. Therefore, the true rate of lymph node involvement remains unknown [45–48]. A global study examined over 4600 patients and found that increasing the extent of lymph node dissection improved the 5-year overall survival rate, especially in T3/T4 patients with 30–4850 lymph nodes involved [49]. The study also stratified patients based on the number of positive lymph nodes (≤ 8 or > 8) and found that those with ≤ 8 positive lymph nodes who underwent thoracic surgery had a significant improvement in survival (64% vs 23%), while no difference was observed in patients with > 8 positive lymph nodes. Extensive lymph node metastasis can indicate subclinical systemic spread, which is why patients with more than eight positive lymph nodes do not benefit from extensive lymph node dissection [34, 50]. Then, the relationship between different esophageal invasion lengths and the metastasis of the periesophageal lymph nodes and when the dissection should be performed remain to be further studied.

This discussion suggests that predicting lymph node metastasis in AEG remains challenging, and the use of the Siewert classification alone is not sufficient to achieve appropriate lymph node dissection. Therefore, new methods of lymph node evaluation are needed for further study to plan the extent of dissection rationally and achieve maximum benefit for patients. In their study, Duan et al. [51] aimed to determine the optimal surgical approach for achieving a more extensive resection of the esophagus and lymph nodes in patients with Siewert type II adenocarcinoma of the esophagogastric junction (AEG). They compared the lengths of proximal margin (PM) that could be achieved with three different surgical approaches: right thoracic segmental esophagectomy (IL), left trans thoracic esophagectomy (LTT), and left thoracoabdominal esophagectomy (LTA). According to the study, the average length of the PM for Ivor-Lewis (IL) esophagectomy using the right transthoracic was 3.8 cm. On the other hand, the average PM lengths for the left thoracic and left thoracoabdominal approaches were 2.4 cm and 1.9 cm, respectively. This means that only the Ivor-Lewis approach was able to achieve satisfactory PM lengths. Although the IL group had the longest operating time, there were no significant differences among the three groups in terms of bleeding volume, tumor size, and other variables that were measured. However, both the IL and LTA groups had more extensive abdominal lymph node dissection than the LTT group. This means that once we determine the optimal PM and lymph node dissection range required for this AEG patient, it can be selected IL, LTT, or LTA based on these data. Certainly, this study is still retrospective, and further prospective randomized controlled trials are necessary for follow-up and validation. Surgeons must confront the risks associated with expanding the surgery to achieve the desired resection and lymph node dissection range. These risks include the possibility of more challenging anastomosis or total gastrectomy with esophagectomy and colon interposition in the lower mediastinum. This surgery carries a higher risk of anastomotic leakage or other postoperative complications [52, 53].

### How to evaluate the length of AEG lower esophageal invasion and periesophageal lymph node metastasis before surgery?

This issue is also a decisive factor in the surgical pathway selection in AEG patients. Currently, there are various methods available to evaluate the extent of invasion in the lower esophagus in AEG. However, endoscopy has its limitations due to the varying degrees of contraction and peristalsis of the digestive tract, as well as the difficulty in identifying the dentate line caused by mucosal displacement, which results in significant measurement errors. On the other hand, 18F-FDG PET/CT lacks effective references and postoperative macroscopic verification, making its clinical application challenging [54]. Esophageal barium meal radiography can magnify the lesion, but it may also lead to an overestimation of its length due to inflammation or food retention at the upper and lower ends of the cancer. On the other hand, it may underestimate the length of the lesion because it cannot observe the extraluminal situation [55]. Enhanced CT and MRI are widely used in the clinical diagnosis of gastric cancer [56, 57], including preoperative evaluation of AEG [58], due to their non-invasiveness and convenience However, these two methods have their own advantages and disadvantages [59], and interference factors may affect the measurement of the length of the lesion [60], such as poor patient cooperation during MRI examination leading to skipping, more artifacts, unstable image quality, and long scan time, while CT is relatively stable. Furthermore, there are numerous factors that impact the determination of invasion length in the lower esophagus in AEG through imaging. These include challenges in distinguishing tumors from surrounding fibrosis and edema, as well as potential interference from respiratory and cardiac cycle artifacts. Additionally, the accuracy of diagnosis is heavily reliant on the clinical experience of radiologists, which can be a time-consuming process with limited sample size accumulation. This can lead to missed diagnoses and misjudgments [61]. Moreover, there exists a discrepancy between the imaging references such as the esophageal hiatus and VCF, and the physiological references like the dentate line and AEG. Consequently, there is a dearth of convenient and efficient techniques for assessing the degree of invasion in the lower esophagus in AEG during clinical practice.

Endoscopic ultrasound (EUS) is established as the most accurate technique for pre-operative locoregional staging of GEJ adenocarcinoma, clearly superior to computed tomography (CT) and magnetic resonance imaging. EUS accuracy for tumor depth (T stage) determination ranges between 85 and 90% [62, 63], while nodal (N) staging accuracy ranges from 70 to 90% [64–66]. However, this high accuracy is concentrated in the N0 stage, where accuracy is significantly reduced for N1, N2, and N3, and after neoadjuvant chemoradiotherapy, the standard EUS staging criteria are inaccurate because chemoradiotherapy-induced changes in inflammation and fibrosis result in thickening and decreased visibility of the five-layered cell wall [67, 68].

Currently, the sentinel lymph node is a viable option for preoperative lymph node assessment. In a preliminary study of 9 patients with adenocarcinoma of the esophagogastric junction (AEG), near-infrared fluorescence imaging was utilized to identify the first lymph node station through indocyanine green (ICG) drainage. The study revealed that in most patients, the first lymph node station was located in the left gastric lymph nodes, with only one patient draining above the diaphragm. In the three patients with lymph node positivity, all had positive lymph nodes at the first station identified by ICG [69]. Other small studies have also shown the potential of this concept, and as more data is gathered, this method may become the standard for lymph node dissection [70, 71].

With the development of artificial intelligence, including deep learning in imaging and recognition in body tissues, it may help us to solve this problem in the near future.

### The value of frozen section analysis in intraoperative margin assessment

Due to the uncertainty surrounding the minimum PM, certain guidelines and review articles recommend the routine use of intraoperative frozen section examination and intraoperative endoscopy to assess the surgical margin. Macroscopic inspection has limitations in predicting negative pathological margins, particularly for tumors with infiltrative growth patterns (indistinct borders, infiltrative, intramural tumors) or tumors with submucosal extension on microscopy [72–74]. Intraoperative frozen section has been found to be highly accurate, sensitive, and specific [17, 19, 36, 73, 75, 76]. It has also been shown to effectively convert positive margins to negative margins, which can expand the scope of surgery.

In the study conducted by Squires et al. [77], 860 cases of AEG were analyzed, and out of these, 520 cases underwent intraoperative frozen section analysis of the proximal margin. Among these cases, 67 were found to be positive. Subsequently, 48 cases underwent additional resection. It is noteworthy that 4.8% (25 cases) of these cases still had positive margins, which is consistent with the rates found in other studies [78]. The study revealed that patients who were converted to the R0 group had a significantly lower local recurrence rate compared to those who remained R1 positive after the final frozen section analysis. However, the attainment of negative margins did not demonstrate a significant correlation with overall survival (OS). Additionally, subgroup analysis based on TNM staging did not reveal a significant difference in RFS or OS between R1 converted to R0 and R1, even in early-stage disease. It is worth noting that only 20% of patients had stage I gastric cancer, and the majority of patients with positive margins were in stages II-III. Kim et al. [79] discovered that patients who were initially R1 positive but underwent additional resection to achieve R0 resection had a lower survival rate compared to those who were initially R0. Similar results have been reported in studies on gastric cancer and margin status recurrence rates [80–82]. If these conclusions are accurate, then the importance of intraoperative frozen section analysis may not be as significant as previously thought.

The research revealed that an intraoperative frozen section is not commonly recommended by pathologists or guidelines for evaluating esophageal margins. This is primarily due to four issues. (1) The technique involves individually freezing, cutting, and staining a significant number of tissue blocks, which results in increased turnover time, resource usage, and manpower costs. (2) There is uncertainty regarding whether the margin should be fully examined to guarantee the accuracy of R0 resection. (3) It is still unclear which high-risk population should be examined. (4) The optimal minimum margin is yet to be determined, and the length of margin required for frozen section cannot be specified. Additionally, false-negative results may occur in clinical applications [78, 83]. Despite advancements in technology, some literature still suggests using intraoperative frozen section analysis for diffuse adenocarcinoma [84]. As a result, additional systematic research is necessary to properly evaluate the pathological margins of AEG.

### Whether neoadjuvant therapy reduce the margin-positive rate in patients with AEG and thus affect the patient's outcome?

Whether neoadjuvant therapy can reduce the positive rate of esophageal margins with locally advanced AEG, there is no sufficient evidence that preoperative use of chemotherapy or chemoradiotherapy can reduce the incidence of positive margins, or improve survival rates [72].

However, in previous studies, it has been found that neoadjuvant therapy has been shown to effectively reduce the size of AEG tumors and the number of metastatic lymph nodes, resulting in downstaging (T and N staging) and ultimately improving the rate of complete tumor resection (R0) [32, 85]. A French trial [86], which included 224 patients with adenocarcinoma (75% with AEG), randomly assigned patients to surgery alone or neoadjuvant chemotherapy (NACT) groups. The rates of R0 resection were 73% and 84% in the two groups, respectively (*p* = 0.04). It is in keeping with the results of the Medical Research Council (MRC) trial, about 75% R0 resection rates [87]. A third phase III trial from the European Organisation for Research and Treatment of Cancer GI group [88] compared preoperative chemotherapy (2 cycles/12 weeks) followed by surgery to surgery alone. Unfortunately, this trial failed to include the 360 required patients and stopped after the inclusion of 144 gastric or AEG adenocarcinoma; After a median follow-up of 4.4 years, no significant increase in OS and a borderline improved recurrence-free survival (*P* = 0.065) were observed despite an improved rate of R0 resection in the preoperative chemotherapy group. From the above studies, NACT can improve the resection rate, but the sample size of each study is small, and the difference from the simple surgery group is not significant. Moreover, the R0 resection rate is also large, and the difference in prognosis is not significant, which needs to be clarified by further large-scale prospective studies.

Similarly, there are considerable differences in R0 resection rates with neoadjuvant chemoradiotherapy(NACRT) compared to surgery alone in various trials. Following NACRT, the R0 resection rate ranges from 81% in the Bosset study [89] to 100% in the Lee study [89, 90]. In studies where patients underwent surgery without neoadjuvant therapy, the R0 resection rate varied from 69% in the Cross trial [32] to 95% in the Lee study [32, 90].

There is currently a lack of conclusive evidence regarding the comparative benefits of NACT and NACRT treatments, particularly in terms of resectability, postoperative morbidity and mortality, histologic tumor response, long-term survival, and health-related quality of life. In Burmeister’s study [91], R0 resection rates were found to be 80.5% and 84.6% for the NACT group and NACRT group, respectively, while in Nusrath’s study [92], they were 86% and 88%. In a phase III trial in Germany [93], 126 of 354 planned patients with AEG adenocarcinoma were divided into preoperative chemotherapy and preoperative chemoradiotherapy groups, with no difference in R0 resection rates (69.5% vs 71.5%). Neoadjuvant chemoradiotherapy (NACRT) has been demonstrated to enhance survival rates in AEG patients [32, 94]. On the basis of these studies’ results, we found that although the R0 resection rate of NACRT was slightly higher than NACT, the difference was not great, even a statistical difference.

Although NACT and NACRT showed an uneven effect on R0 resection rate in different studies, it was still a significant improvement compared with the surgery-alone group. Most patients with locally advanced disease receive chemoradiotherapy or chemotherapy alone and it could reduce the number of lymph nodes, the relationship between re-staging and outcome remains unclear due to the change in the ratio of positive to total lymph node metastasis [95], so there is no effective method to judge the impact of neoadjuvant therapy on overall prognosis. The question of whether neoadjuvant therapy should be widely utilized in patients with AEG remains uncertain and necessitates larger randomized studies for clarification (Table 4).

**Table 4:** Changes in R0 resection rates after neoadjuvant therapy

Study	*N* (AEG)	R0 resection rates			
		Surgery alone	NACT	NACRT	*P* value
FNCLCC [86]	168	73%	84%		0.04
MRC trial [87]	206	76.4%	75%		/
European Organisation [88]	144	66.7%	81.9%		0.036
Bosset study [89]	297			81%	/
Lee study [89, 90]	101	95%		100%	/
Cross trial [32]	275	69%		92%	< 0.001
Burmeister’s study [91]	75		80.5%	84.6%	0.61
Nusrath’s study [92]	70		86%	88%	1
Germany trial [93]	126		69.5%	71.5%	/

Adverse prognostic factors for advanced-stage SRC include the invasive SRC phenotype, a high risk of lymph node and peritoneal metastasis, adjacent organ involvement, differential response to neoadjuvant therapy, and a low R0 resection rate [96]

Adverse prognostic factors for advanced-stage SRC include the invasive SRC phenotype, a high risk of lymph node and peritoneal metastasis, adjacent organ involvement, differential response to neoadjuvant therapy, and a low R0 resection rate [96].

The study showed that SRC may not respond as well to radiation and chemotherapy. Then there is ongoing debate whether neoadjuvant therapy or upfront surgery is the preferred treatment approach. SRC patients are more likely to have positive surgical margins, and that after NACRT, they experience less pathological downstaging compared to AC patients [97]. The French FREGAT study [98] discovered that neoadjuvant chemotherapy had a limited impact on the metastatic potential and T/N staging of SRC. Chirieac et al. [99] discovered that the survival time of SRC patients who only underwent surgery was relatively low. On the other hand, the survival time after neoadjuvant therapy was significantly better than that of AC patients. Despite ongoing debate, we recommend that SRC not be excluded from neoadjuvant chemotherapy as some patients may still benefit. There is currently insufficient evidence to support the notion that initial surgical resection leads to better outcomes. Moving forward, the studies should focus on stratifying patients by SRC component to gain a deeper understanding of the effects of neoadjuvant therapy.

Is neoadjuvant radiotherapy a superior option to chemotherapy for achieving improved local control in AEG SRC? Studies have shown that after NACT, there were more R1/R2 resections in SRC, which confirmed its correlation with poorer local RFS. It appears that NACRT may offer more benefits for SRC patients, particularly in terms of local–regional control. As a result, additional research on the efficacy of intensified local regional treatment is necessary [100]. At present, the majority of studies pertaining to this subject are retrospective in nature, and there is a possibility of bias due to patient selection. However, it is worth noting that NARCT stands out as the only prognostic factor that has been determined through multivariate analysis. Despite its limitations, NARCT still holds some value in guiding clinical practice.

There are several important issues that need to be addressed to further explore the margin distance in AEG: (1) determining the optimal distance for PM is a complex task that requires conducting large prospective clinical studies. Such studies should consider various factors such as the length of tumor invasion and the patient’s clinical characteristics to identify the optimal PM distance for each patient. (2) Preoperative assessment of the extent of tumor invasion into the lower esophagus is crucial in selecting the appropriate length of the esophagus to be resected, as well as determining the optimal PM distance. This assessment helps to tailor the surgical procedure to the patient’s specific needs and ensures that the appropriate amount of tissue is removed to achieve the best possible outcome. (3) Clarifying the choice of surgical approach before the procedure is essential for achieving the best surgical outcome. This will not only reduce the incidence of unexpected surgical complications but also improve patient management and prognosis. (4) A deeper understanding of the clinical and biological characteristics of AEG, particularly signet ring cell carcinoma, is crucial. This includes examining the clinical features of esophageal invasion and lymph node metastasis. (5) Optimizing the preoperative neoadjuvant therapy strategy by formulating treatment standards that will effectively downstage the tumor is important. This will help to achieve the best possible postoperative margin by reducing the extent of surgery required. Overall, addressing these issues will help to improve the management and outcomes of patients with AEG.

## Data Availability

All data generated or analyzed during this study are included in this published article.
